# Sponyloarthritis features forecasting the presence of HLA-B27 or sacroiliitis on magnetic resonance imaging in patients with suspected axial spondyloarthritis: results from a cross-sectional study in the ESPeranza Cohort

**DOI:** 10.1186/s13075-015-0779-y

**Published:** 2015-09-23

**Authors:** Victoria Navarro-Compán, Eugenio de Miguel, Désirée van der Heijde, Robert Landewé, Raquel Almodóvar, Carlos Montilla, Emma Beltrán, Pedro Zarco

**Affiliations:** Department of Rheumatology, IdiPaz, University Hospital La Paz, Madrid, Spain; Department of Rheumatology, Leiden University Medical Center, Leiden, The Netherlands; Department of Clinical Immunology & Rheumatology, Academic Medical Center, University of Amsterdam, Amsterdam, The Netherlands; Department of Rheumatology, Atrium Medical Center Heerlen, Heerlen, The Netherlands; Department of Rheumatology, University Hospital Foundation Alcorcón, Madrid, Spain; Department of Rheumatology, Hospital Clínico Universitario de Salamanca, Salamanca, Spain; Department of Rheumatology, Hospital Universitario General de Valencia, Valencia, Spain

## Abstract

**Introduction:**

Chronic back pain (CBP) is frequently the presenting symptom in patients with suspected axial spondyloarthritis (axSpA). Presence of sacroiliitis on magnetic-resonance-imaging (MRI) or HLA-B27 adds to diagnostic certainty. However, these costly tests cannot be applied in all patients with CBP. This study aims to investigate which SpA features increase the likelihood of a positive HLA-B27 or positive MRI of the sacroiliac-joints (MRI-SI) in patients with suspected axSpA.

**Methods:**

Data from 665 patients with CBP within the ESPeranza Programme were analysed. Diagnostic utility measures (LR+, LR−) for a positive MRI-SI or HLA-B27 were calculated for various definitions of inflammatory back pain (IBP), their separate items and for other SpA features.

**Results:**

Pretest probabilityies of a positive result was 41 % for MRI-SI and 40 % for HLA-B27. For a positive MRI-SI result the most useful IBP characteristic was alternating buttock pain (LR + =2.6). Among the IBP-criteria, fulfillment of the ‘ASAS criteria’ (LR + =2.1) was most contributory. Interestingly, the addition of alternating buttock pain to the Calin/ASAS-IBP criteria (LR + =6.0 and 5.5, respectively) or the addition of awakening at second half of night to the Calin-IBP criteria (LR + =5.5) increased the pre-test probability of MRI-sacroiliitis from 41 % to 79–80 %. Dactylitis (LR + =4.1) and inflammatory bowel disease (IBD) (LR + =6.4) increased this probability to 73 % and 81 %, respectively. To forecast HLA-B27 positivity, awakening at the second half of the night, fulfillment of the ASAS-IBP definition and uveitis were the most useful, but only marginally predictive (LR + = 1.3, 1,6 and 2.6, respectively).

**Conclusions:**

If patients with suspected axial SpA have either 1) IBP according to Calin/ASAS definition plus alternating buttock pain, or 2) IBP according to Calin definition plus awakening at night, or 3) dactylitis or 4) IBD, the probability of finding a positive MRI-SI increases significantly.

## Introduction

Axial spondyloarthritis (axSpA) has a major impact on physical function and quality of life [1]. Nevertheless, despite these important consequences patients with axSpA have been traditionally diagnosed after several years of symptoms [2]. In this sense, magnetic resonance imaging of sacroiliac joints (MRI-SI) has become important in the last decade, especially in the early stages of the disease. Nowadays, performing imaging, and testing human leucocyte antigen B27 (HLA-B27) are among the most important diagnostic procedures in patients with (suspicion of) axSpA. Accordingly, imaging and HLA-B27 results are also the entry criterion for classifying patients with chronic back pain (CBP) as having axSpA based on the classification criteria of the Assessment of SpondyloArthritis International Society (ASAS) [3].

Furthermore, in patients with suspected axSpA the starting point of the disease is usually the presence of CBP. However, CBP is one of the most prevalent symptoms in the general population and therefore it is essential to be able to select which patients with CBP have the highest chance of being diagnosed with axSpA. For this purpose, several referral strategies including different manifestations at the beginning of the disease have been developed for primary physicians, but [4–6] in clinical practice most patients are referred from primary care to rheumatologists because of inflammatory back pain (IBP) [4]. However, despite the publication of several IBP definitions [7–9], there is only poor agreement between primary physicians and rheumatologists about the presence of IBP. In addition to this, the original algorithm for diagnosing axSpA has been recently modified, excluding IBP as an obligatory entry criterion. This modification has been applied based on the finding that up to 30 % of patients with axSpA do not have IBP, and therefore its inclusion as an obligatory entry criterion results in too many misdiagnoses [10]. According to the modified algorithm, complementary examinations (HLA-B27 and MRI) need to be considered in those patients without sacroiliitis on conventional x-rays or with fewer than four featuresof SpA, which usually comprises most of the referred patients. But in clinical practice, due to efficiency reasons these tests must usually be restricted to patients with higher probability of a positive result. Based on clinical features, it would be very helpful for rheumatologists to identify which referred patients have the highest likelihood of ultimately being diagnosed as having axSpA. Such features of SpA that can be obtained by taking history or by simple physical examination, could potentially contribute to an efficient test-sequence to be applied in patients presenting with CBP and to optimise the use of supplementary tests in these patients. Based on this, this study aims to investigate which features of SpA may increase the pre-test probability of a positive test result of HLA-B27 or MRI-SI in patients with suspected axSpA, which is a step forward in making a diagnosis of axSpA.

## Methods

### Study design and population

This study was performed within the context of the ESPeranza Programme. The details from this initiative have been previously reported [11–13]. In summary, the ESPeranza Programme is a Spanish prospective multicentre national health programme aiming to facilitate early diagnosis of patients with spondyloarthritis. The programme was designed in compliance with the Helsinki Declaration and approved by the Ethical Committee of Research Unit of Hospital Reina Sofía in Córdoba. Over 3 years from April 2008, primary physicians and other specialists were asked by rheumatologists to refer patients meeting the following criteria: 1) age from 18 to 45 years; 2) symptom duration between 3 and 24 months; and 3) fulfilling one of the following three symptoms: IBP, asymmetrical arthritis or spinal/joint pain plus the presence of at least one of the following features: psoriasis, inflammatory bowel disease (IBD), anterior uveitis, radiographic sacroiliitis, HLA-B27 positivity, or a family history of spondylitis. All patients provided signed informed consent. In total, 25 centres across the country participated in the programme and 775 patients met the inclusion criteria. For the current study, only those patients referred with axial symptoms (n = 665) were selected.

### Data collection and clinical measures

In the ESPeranza programme, sociodemographic and disease data were collected including age, sex, symptom duration, family history of SpA or related diseases, peripheral manifestations (enthesitis, dactylitis and/or arthritis) and extra-articular manifestations (psoriasis, uveitis, IBD). In addition, all typical characteristics of IBP were separately collected including: morning stiffness, improvement of back pain with exercise and not with rest, alternating buttock pain, insidious onset and awakening during the second half of the night. Moreover, the SpA feature related with CBP and CBP, “Good response to nonsteroidal anti-inflammatory drugs (NSAIDs)” were collected too. Based on this information, the fulfilment of the different existing IBP criteria was established as follows: Calin (at least four out of the following five criteria: age <40 years, insidious onset, duration >3 months, morning stiffness, improvement with exercise); Berlin (at least two out of the following four: morning stiffness, improvement with exercise and not with rest, awakening during the second half of the night, alternating buttock pain) and ASAS (at least four out of the following five: age <40 years, insidious onset, improvement with exercise, no improvement with rest, night pain).

Clinical assessments were also performed, including disease activity (Bath ankylosing spondylitis disease activity index, BASDAI), function (Bath ankylosing spondylitis functional index, BASFI), and laboratory tests (C-reactive protein (CRP)). The presence of HLA-B27 was tested in the local laboratory of each centre according to the standard procedure. The evaluation of conventional radiographs of the cervical and lumbar spine and of the pelvis was part of the protocol. MRI-SI was not included in the protocol as mandatory. Nevertheless, all rheumatologists were asked to perform MRI-SI if possible. However, due to existing differences between centres in accessibility to MRI, including budget limitations and waiting lists for MRI, MRI-SI was not performed all patients. MRI-SI was evaluated locally by one reader at each hospital, who evaluated the presence or absence of sacroiliitis according to the ASAS definition [14].

### Statistical analysis

For descriptive purpose, results for continuous variables are presented as mean and standard deviation (SD) and for categorical variables are shown as percentage and relative frequencies. The diagnostic utility of each IBP characteristic, good response to NSAIDs, IBP definitions (Calin, Berlin, ASAS) and other features of SpA for a positive MRI-SI or HLA-B27 was calculated. Diagnostic utility was assessed based on sensitivity, specificity, positive and negative predictive values (PPV and NPV) and especially, positive and negative likelihood (LR+ and LR−). Importantly, a cutoff value of 4.0 was used for LR+ to define a feature of SpA as useful. The reason for the selection of this cutoff is that it has been associated with a moderate increase (approximately 25 %) in the likelihood of disease [15]. Finally, post-test probability was calculated based on the positive and negative likelihood ratios using the following conversion formula:

Odds = Probability/(1 – Probability).

According to Bayes’ law (post-test odds = pre-test odds* LR, in which LR– is used if a feature is absent and the LR+ if a feature is present) the likelihood of finding a positive MRI/HLAB27 was estimated in patients with this particular feature present or absent.

## Results

### Baseline characteristics

A total of 665 patients with axial symptoms were referred and included in ESPeranza. Fifty five percent were male, mean (SD) age was 33.2 (7.1) years and mean (SD) symptom duration was 12.6 (6.6) months. For most of them (n = 653; 98.2 %) data was also available on HLA-B27 testing, and these were included in the analysis to evaluate the association between IBP characteristics and the presence of HLA-B27. Approximately half of the patients (n = 326; 49 %) had MRI-SI performed and were included in the analysis to investigate the association between IBP characteristics and positive MRI-SI. Demographic and disease characteristics were similar for patients in the whole cohort compared with patients in whom MRI-SI had been performed (Table 1). In total, 270 (41 %) patients were HLA-B27 positive and 130 (40 %) patients had a positive MRI-SI result (pre-test probabilities).

**Table 1:** Baseline characteristics for all patients with axial symptoms included in Esperanza (left column) and for the subgroup of patients who underwent MRI-SI (right column)

Characteristic	Patients with axial symptoms referred to Esperanza (n = 665)	Patients with axial symptoms and SIJ MRI available (n = 326)
Age, years	33.2 ± 7.1	32.8 ± 7.0
Male	363 (54.6)	175 (53.7)
Symptoms duration, months	11.9 ± 6.6	12.6 ± 6.4
Morning stiffness	393 (59.1)	195 (59.8)
Improve with exercise, not with rest	208 (31.3)	97 (29.8)
Alternating buttock pain	197 (29.6)	98 (30.1)
Insidious onset	430 (64.7)	256 (78.5)
Awakening at second half of night	315 (47.4)	169 (51.8)
Response to NSAIDs	407 (61.2)	200 (61.3)
Enthesitis	107 (16.1)	45 (13.8)
Psoriasis	82 (12.3)	35 (10.7)
Dactylitis	26 (3.9)	14 (4.3)
IBD	26 (3.9)	10 (3.1)
Uveitis	34 (5.1)	16 (4.9)
Arthritis	95 (14.3)	37 (11.3)
Family history	170 (25.6)	89 (27.3)
HLA-B27	270 (40.6)	144 (44.2)
Elevated CRP, mg/L	177 (26.6)	89 (27.3)
BASDAI	4.0 ± 2.3	3.9 ± 2.3
BASFI	2.5 ± 2.4	2.3 ± 2.4
ASAS criteria for axial SpA	291 (43.8)	167 (51.2)
Imaging arm	194 (29.2)	132 (40.5)
MRI-SI positive	85 (12.8)	85 (26.1)
mNY-positive^a^	109 (16.4)	47 (14.4)
Clinical arm only	97 (14.6)	35 (10.7)

Results are presented as mean ± SD or number (%). ^a^Patients who were both magnetic resonance imaging of sacroiliac joints (MRI-SI)-positive and modified New York criteria for ankylosing spondylitis (mNY)-positive) are included. *NSAIDs* nonsteroidal anti-inflammatory drugs, *IBD* inflammatory bowel disease, *HLA-B27* human leucocyte antigen B27, *CRP* C-reactive protein, *BASDAI* Bath ankylosing spondylitis disease activity index, *BASFI* Bath ankylosing spondylitis functional index, *ASAS* Assessment of SpondyloArthritis International Society, *SpA* spondyloarthritis

### IBP characteristics forecasting the presence of sacroiliitis on MRI or HLA-B27

Table 2 shows the results for the diagnostic utility of these characteristics. While alternating buttock pain was most contributory in predicting a positive MRI-SI result (LR + = 2.6; LR − = 0.6), awakening during the second half of the night was the most contributory in predicting a positive HLA-B27 result (LR+ =1.3; LR − = 0.8). However, individually none of the characteristics made a very strong contribution.

**Table 2:** Values for diagnostic utility measures for each of the inflammatory back pain characteristics in relation to the presence of sacroiliitis on MRI, and positive HLA-B27 testing

	Sacroiliitis on MRI						HLA-B27					
	Sen	Spe	LR+	LR−	PPV	NPV	Sen	Spe	LR+	LR−	PPV	NPV
Morning stiffness for >30 minutes	68.5	45.9	1.3	0.7	45.6	68.7	63.3	43.6	1.1	0.8	44.2	62.8
Improvement with exercise, not with rest	31.5	71.4	1.1	0.1	42.3	61.1	33.7	70.2	1.1	0.9	44.4	60.0
Alternating buttock pain	47.7	81.6	2.6	0.6	63.3	70.2	31.9	71.3	1.1	0.9	43.9	59.7
Insidious onset	87.7	27.6	1.2	0.4	44.5	77.1	68.1	37.1	1.1	0.9	43.3	62.3
Awakening in second half of the night	64.6	56.6	1.5	0.6	49.7	70.7	55.2	57.4	1.3	0.8	47.8	64.5
Response to NSAIDs	69.2	43.9	1.2	0.7	45.0	68.3	67.4	43.3	1.2	0.8	45.6	65.4

*MRI* magnetic resonance imaging, *HLA-B27* human leucocyte antigen B27, *Sen* sensitivity, *Spe* specificity, *LR+* positive likelihood ratio, *LR−* negative likelihood ratio, *PPV* positive predictive value, *NPV* negative predictive value, *NSAIDs* nonsteroidal anti-inflammatory drugs

### IBP definitions forecasting the presence of sacroiliitis on MRI or HLA-B27

The three multi-item IBP definitions performed more or less similarly in terms of a LR+. To forecast a positive MRI-SI, the ASAS criteria performed slightly better but still produced very low LRs (LR + = 2.1; LR − = 0.7) (Table 3). Based on these results, it was decided to assess whether or not the addition of other IBP characteristics (or good response to NSAID) which were not included in the definitions of IBP increased their diagnostic utility. This means that patients had to fulfil the specific IBP definition plus the additional characteristic. The results for this analysis are depicted in Table 3. Of all possible combinations, the ones that performed better were the combination of the Calin/ASAS definition and alternating buttock pain (LR + = 6.0 and 5.5, respectively; LR − = 0.7 for both combinations) and the combination of the Calin definition and awakening in the second half of the night (LR + = 5.5; LR − = 0.8). These combinations had a post-test probability of sacroiliitis on MRI of 79–80 % if the combined criterion was present, and 32–35 %, if the combined criterion was absent.

**Table 3:** Values for diagnostic utility measures for each of the inflammatory back pain definitions and other features of SpA in relation to the presence of sacroiliitis on MRI and positive HLA-B27 testing

	Sacroiliitis on MRI						HLA-B27					
	Sen	Spe	LR+	LR−	PPV	NPV	Sen	Spe	LR+	LR−	PPV	NPV
IBP definition												
Calin criteria	51.5	70.9	1.8	0.7	54.0	68.8	36.3	74.2	1.4	0.9	49.7	62.3
Berlin criteria	72.3	50.5	1.5	0.6	49.2	73.3	64.1	49.3	1.3	0.7	47.1	66.1
ASAS criteria	47.7	77.0	2.1	0.7	57.9	68.9	31.5	80.7	1.6	0.9	53.5	62.3
IBP definition plus other IBP characteristics												
Calin + alt. buttock	30.8	94.9	6.0	0.7	80.0	67.4	17.4	86.4	1.3	1.0	51.0	56.3
Calin + NSAIDs	36.2	80.1	1.8	0.8	54.7	65.4	28.5	75.7	1.2	0.9	48.8	56.5
Calin + night	28.0	94.9	5.5	0.8	77.8	67.4	16.2	87.9	1.3	1.0	52.3	56.3
Calin + alt. buttock + night	26.9	94.9	5.3	0.8	77.8	66.2	16.0	88.1	1.3	1.0	52.3	56.3
Calin + alt. buttock + NSAIDs	20.0	96.9	6.5	0.8	81.3	64.6	10.4	91.0	1.2	1.0	48.4	55.5
Calin + Night + NSAIDs	29.2	87.2	2.3	0.8	60.3	65.0	21.5	88.3	1.8	0.9	56.3	61.5
Berlin + Insidious	64.6	62.2	1.7	0.6	53.1	72.6	50.7	53.1	1.1	0.9	46.8	57.0
Berlin + NSAIDs	53.8	68.4	1.7	0.7	53.0	69.1	48.9	66.1	1.4	0.8	50.4	64.7
Berlin + insidious + NSAIDs	47.7	76.0	2.0	0.7	56.9	68.7	34.0	67.2	1.0	1.0	45.8	55.6
ASAS + alt. Buttock	30.8	94.4	5.5	0.7	78.4	94.4	18.1	86.4	1.3	0.9	52.0	56.5
ASAS + NSAIDs	36.9	86.2	2.7	0.7	64.0	67.3	24.4	82.0	1.4	0.9	56.4	61.9
ASAS + stiffness	39.2	81.1	2.1	0.7	58.0	66.8	31.3	76.3	1.3	0.9	51.7	57.7
ASAS + alt. Buttock + NSAIDs	21.5	96.4	6.0	0.8	80.0	64.9	10.7	94.3	1.9	0.9	56.9	94.3
ASAS + stiffness + NSAIDs	28.5	88.8	2.5	0.8	62.7	65.2	19.4	83.1	1.1	1.0	48.3	55.9
ASAS + stiffness + alt. buttock	26.9	94.9	5.3	0.8	77.8	66.2	16.0	88.1	1.3	1.0	52.3	56.3
Other SpA features												
Enthesitis	11.5	84.7	0.8	1.0	33.3	59.1	17.8	85.1	1.2	0.9	85.1	45.7
Peripheral arthritis	13.8	90.3	1.4	0.9	48.6	61.2	16.7	87.7	1.4	0.9	87.7	48.9
Dactylitis	23.1	94.4	4.1	0.8	21.4	59.3	4.8	96.9	1.6	09	96.9	52.0
Uveitis	6.2	95.9	1.5	0.9	50.0	60.6	8.1	96.9	2.6	0.9	96.9	64.7
Psoriasis	10.0	88.8	0.9	1.0	37.1	59.8	7.0	83.8	0.4	1.1	83.8	23.5
Inflammatory bowel disease	23.1	96.4	6.4	0.8	30.0	59.8	1.5	94.5	0.3	1.0	94.5	16.0
Family history of SpA	27.7	73.0	1.0	0.9	40.4	60.3	33.7	80.2	1.7	0.8	80.2	54.5
Elevated CRP	40.8	77.5	1.8	0.8	55.1	66.0	38.5	77.1	1.7	0.8	77.1	54.6
HLA-B27	59.7	65.1	1.7	0.6	53.5	70.6	–	–	–	–	–	–

*MRI* magnetic resonance imaging, *HLA-B27* human leucocyte antigen B27, *Sen* sensitivity, *Spe* specificity, *LR+* positive likelihood ratio, *LR−* negative likelihood ratio, *PPV* positive predictive value, *NPV* negative predictive value, *ASAS* Assessment of SpondyloArthritis International Society, *Alt.* alternating, *NSAIDs* nonsteroidal anti-inflammatory drugs, *SpA* spondyloarthritis, *CRP* C-reactive protein

Similar to sacroiliitis, the three IBP definitions performed similarly well to predict a positive HLA-B27 result (Table 3). The ASAS criteria performed best but the LR+ (1.6) and LR− (0.9) was poor. On the other hand, and in contrast to the results for sacroiliitis, the addition of other IBP characteristics (or good response to NSAIDs) to the existing IBP definitions only slightly increased their diagnostic utility.

### Other features of SpA forecasting the presence of sacroiliitis on MRI or HLA-B27

The utility of features of SpA other than IBP in helping to anticipate a positive (or a negative) MRI-SI or a positive (or negative) HLA-B27 test result was also evaluated. These SpA features included peripheral and extra-articular manifestations, family history of SpA or related diseases, elevated CRP and HLA-B27 (only for MRI-SI). While dactylitis and IBD appeared to be the most useful features of SpA for forecasting positive MRI-SI (LR + = 4.1 and 6.4, respectively), uveitis was the best for a positive HLA-B27 test, but had a LR+ of only 2.6 (Table 3). The presence or history of dactylitis or IBD resulted in a post-test probability for positive MRI of 73 % and 81 %, respectively, compared with the pre-test probability of 40 %. The post-test probability decreased from 40 % to 35 % if both dactylitis and IBD were confirmed absent.

## Discussion

This study evaluates the utility of IBP characteristics, separately and combined, as well as other features of SpA to anticipate the presence of sacroiliitis on MRI or a positive result in HLA-B27 testing in patients with suspected axSpA.

In contrast to most prediction analyses, we have adopted a Bayesian approach based on likelihood ratios, rather than a ‘frequentist approach’ with odds ratios focusing on positive test results. The Bayesian approach is far closer to clinical reality because it directly visualizes the consequences of a positive test result (here, IBP criteria present) and a negative test result (here, IBP criteria absent) on the likelihood of the outcome (here, positive MRI-SI result) in a cohort with a given prevalence of the outcome. In contrast, the effects of odds ratios obtained with regular regression analysis (either significant or not) on the outcome of interest are entirely dependent on the prevalence of the outcome, and the clinical value is difficult to disentangle (and often disappointingly low).

To predict the presence of sacroiliitis on MRI, we have found that alternating buttock pain and the ASAS-definition for IBP are the most useful characteristic and criterion, respectively, but neither of them performed sufficiently well when tested alone. Interestingly, the addition of the criterion of alternating buttock pain to the Calin or ASAS definition increased the positive likelihood ratio for MRI-SI notably compared to the Calin or ASAS definition alone (LR+ 6.0 vs 1.8 respectively for the Calin definition and 5.5 vs 2.1, respectively, for the ASAS definition). When the Berlin algorithm had been designed, the criterion of alternating buttock pain also increased the specificity for axSpA but at the cost of a very low sensitivity (because alternating buttock pain was a rare finding) [16]. When the ASAS definition for IBP was later developed, it therefore did not contribute independently to the presence of IBP according to expert opinion and therefore was not included in the proposed definition [7]. In the current study, we decided to re-examine the performance of the criterion of alternating buttock pain, because of the high cost associated with MRI-SI: we were searching for a criterion that could help us in deciding which patients should undergo MRI-SI. Moreover, the addition of the IBP characteristic of awakening in the second half of the night to the Calin criteria also increased significantly the likelihood ratio of having a positive MRI-SI compared to the performance of the Calin criteria alone (LR+ 5.5 vs 1.8, respectively). Furthermore, in addition to IBP, other features of SpA have been identified for predicting the presence of sacroiliitis on MRI in patients with suspected axSpA. The most useful features we observed were dactylitis and IBD.

In addition, in order to recommend a tool as diagnostically relevant in the context of a Bayesian approach, both positive and negative LR should be explored [15]. It is obvious that single features fall short in this regard. But if a patient with CBP has any of the four following characteristics: 1) IBP according to the Calin or ASAS definition plus alternating buttock pain; 2) IBP according to the Calin definition plus awakening in the second half of the night; 3) dactylitis; or 4) IBD, the probability of having a positive MRI-SI is very high (73–81 %). So in the case of limited resources, the presence of any of these three characteristics may help to increase the diagnostic efficiency of the MRI-SI.

Forecasting a positive HLA-B27 test result is more difficult: neither the individual IBP characteristics, nor the existing or other possible combinations were found useful to predict a positive HLA-B27 test. Among other SpA features, uveitis appeared to be the most closely related to a positive HLA-B27, but it is not sufficiently useful.

To our knowledge, this is the first study investigating this topic. This study has a few strengths: first, many patients with suspected axSpA were included from all over Spain. Second, the mean symptom duration in these patients was very short compared to most diagnostic utility studies and this is an advantage in the referral of patients to early axSpA clinics. Most importantly, patients included in this analysis came from a national health programme representing a typical scenario of common clinical practice that involved primary physicians, rheumatologists and other specialists who usually take care of patients with suspected axSpA. Relying on the conclusions of a local reader (radiologist or rheumatologist) rather than on a report of a central reading committee (as in clinical trials) should also be interpreted in this context, but this is exactly the clinical situation that we wanted to address.

Limitations should be considered too: the most important limitation is the possibility of confounding by indication. For the analysis of the association between IBP characteristics, or other features of SpA, and the presence of sacroiliitis on MRI, data were only included for those patients in whom MRI-SI was ordered by the rheumatologist. As there is always a reason to do so, patient selection is very likely, which may have implications for the external validity of this study. On the other hand, patients included in this analysis (because they had MRI ordered) had similar characteristics to the overall group of patients. Second, inter-reader variation by 25 different local readers who assessed the MRI may have influenced this study too. In this sense, it can be argued that the results of this study are applicable to clinical practice where many different readers are usually involved. Third, all patients included in this study came from the same country and the same cohort, which may limit the extrapolation of the results to other countries. However, in a sub-analysis of Spanish patients participating in the international study evaluating several referral strategies for diagnosis of axSpA, the national results were very similar to those observed in the global population [17]. Fourth, It should be pointed out that the diagnostic performance is dependent on the prior probability of the outcome of interest: While the diagnostic performance in terms of likelihood ratios is considered to be rather stable across cohorts, the application of these likelihood ratios in cohorts with other prior probabilities are very different. A final limitation is obviously that we failed to frame a group of patients in whom the likelihood of a positive MRI and/or positive HLAB27 was very low, as the negative likelihood ratios of all features tested fell short as the lowest LR− was 0.6.

## Conclusions

In summary, if a patient has IBP according to the Calin or ASAS definition plus alternating buttock pain or IBP according to the Calin criteria plus awakening in the second half of the night, or dactylitis or IBD, the probability of positive MRI-SI increases from 40 to approximately 75 %. So in the case of limited resources, the presence of any of these four characteristics may improve the efficiency of ordering MRI in patients with suspected axSpA. None of the IBP definitions or any of the other SpA features appear to be useful to forecasting a positive HLA-B27 test.

## Abbreviations

ASAS: Assessment of SpondyloArthritis International Society
AxSpA: axial spondyloarthritis
BASDAI: Bath ankylosing spondylitis disease activity index
BASFI: Bath ankylosing spondylitis functional index
CBP: chronic back pain
CRP: C-reactive protein
HLA-B27: Human leucocyte antigen B27
IBD: inflammatory bowel disease
IBP: inflammatory back pain
LR−: negative likelihood ratio
LR+: positive likelihood ratio
MRI-SI: magnetic resonance imaging of sacroiliac joints
NPV: negative predictive value
NSAID: nonsteroidal anti-inflammatory drug
PPV: positive predictive value
